# Attenuation of Hyperglycemia in Diabetic Rats Assisted by Immobilized Probiotic in Sodium Alginate

**DOI:** 10.1007/s12602-023-10166-3

**Published:** 2023-10-10

**Authors:** José J. Arriaga-Morales, Cynthia Ordaz-Pichardo, Roberto Castro‑Muñoz, Enrique Durán-Páramo

**Affiliations:** 1 Laboratorio de Bioconversiones, Unidad Profesional Interdisciplinaria de Biotecnología, Instituto Politécnico Nacional, Av. Acueducto s/n, Col. La Laguna, Gustavo A. Madero, 07340 CDMX Mexico; 2 Laboratorio de Biología Celular y Productos Naturales, Escuela Nacional de Medicina y Homeopatía, Instituto Politécnico Nacional, Guillermo Massieu Helguera 239, Col. La Escalera, Gustavo A. Madero, 07320 CDMX Mexico; 3 Tecnologico de Monterrey, Campus Toluca. Av. Eduardo Monroy Cárdenas 2000 San Antonio Buenavista, 50110 Toluca de Lerdo, Mexico; 4 Department of Sanitary Engineering, Faculty of Civil and Environmental Engineering, Gdansk University of Technology, G. Narutowicza St. 11/12, 80 – 233, Gdansk, Poland

**Keywords:** Cell immobilization, *Lactobacillus casei*, Probiotic, Diabetes, Antihyperglycemic effect, Murine model

## Abstract

Diabetes mellitus type 2 (DM2) is the most common chronic disease worldwide, characterized mainly by increased glucose concentration in the blood and affecting several organs’ functionality. The daily consumption of probiotic bacteria can help control diabetes and reduce the damage caused. Cell immobilization techniques are a powerful tool that provides physical cell protection to such probiotic bacteria against gastrointestinal conditions. We suggest that cell immobilization could be a significant vector for delivering a high quantity of viable probiotics to the gut, helping attenuate hyperglycemia in diabetic rats. Seventy male Wistar rats were used in this work. Nicotinamide was administrated via intraperitoneal injection 15 minutes before inducing type 2 diabetes (DM2), followed by a second intraperitoneal injection of streptozotocin to induce DM2. Rats were divided into seven groups. For 45 days, a specific treatment was applied to each group. The group of rats, supplied with immobilized *Lactobacillus casei,* showed a serum glucose concentration of 137 mg/dL, which was close to the one observed in the groups of healthy rats (117 mg/dL) and rats treated with metformin (155 mg/dL). The diabetic rats without treatment presented a higher serum glucose concentration (461 mg/dL). In the rats treated with immobilized *L. casei*, there was no biochemical parameter alteration, and the cell morphology of the analyzed tissues was similar to those of the healthy group. The consumption of immobilized *L. casei* could allow a high quantity of viable probiotics to be delivered to the gut, reducing serum glucose concentration by up to 70% compared to diabetic rats and reducing organ damage caused by diabetes.

## Introduction

Diabetes mellitus (DM) is a degenerative metabolic disorder, representing one of the leading causes of mortality and morbidity worldwide. By 2045, about 578 million people worldwide will suffer from this disease [1, 2]. The development of DM2 has been linked to an intestinal microbiota imbalance; apparently, some intestinal pathogenic microorganisms trigger inflammatory processes that reduce insulin sensitivity and stimulate the immune response that destroys the β-cells in the pancreas [3–9]. An alternative to restoring the intestinal microbiota is the consumption of probiotics from the genera *Lactobacillus* and *Bifidobacterium*. When ingested in adequate quantities, these bacteria benefit the host; probiotics compete with pathogenic microorganisms for adhesion sites and nutrients in the colon. However, they must remain viable during transit in the gastrointestinal system [10–12]. Cell immobilization techniques are a powerful tool that provides physical cell protection against digestive conditions [13–15]. Therefore, this study aims to demonstrate the effect of immobilized cells of *Lactobacillus casei* subsp. *casei*, which was used as a diet complement in diabetic rats. We suggest that cell immobilization could be a significant vector for delivering a high quantity of viable probiotics to the gut, which could help attenuate the DM2 complications.

## Materials and Methods

### Culture of *Lactobacillus casei* and Obtention of Free and Immobilized Cells

*Lactobacillus. casei subsp. casei* NRRL-1922 (*L. casei*) was donated by the Department of Agriculture of the USA. The strain was cultivated in MRS medium for 24 h at 37 °C and 120 rpm; the culture was then centrifuged at 6000 rpm for 10 min at 4 °C; the biomass concentration recovered was 10^12^ CFU/100 μL, which was suspended in phosphate-buffered saline (PBS). The cell immobilization procedure was carried out under aseptic conditions, as follows: The biomass from a liquid culture of *L. casei* previously incubated for 24 h at 37 °C was recovered by centrifugation at 6000 rpm for 10 min at 4 °C. The biomass was suspended in a 2% sodium alginate solution (Sigma-Aldrich). Subsequently, using a peristaltic pump adapted with an injector hose, the *L. casei* cell suspension in sodium alginate was added dropwise with gentle agitation into a CaCl_2_ solution (0.3 M). Each drop of the *L. casei* cell suspension in sodium alginate formed a microparticle of calcium alginate gel upon contact with the CaCl_2_ solution [16]. Spherical calcium alginate microparticles with a diameter of 2.1 ± 0.08 mm containing 10^12^ CFU/100 μL gel of *L. casei* were obtained from the cell immobilization process. Once the microparticles were formed, they were quantified, and it was established that 100 μL of sodium alginate containing the biomass formed 12 spherical microparticles containing a total of 10^12^ CFU of *L. casei*.

### Evaluation of the Viability Loss of *Lactobacillus casei* Under In Vitro Simulated Gastrointestinal Conditions

Free and immobilized cells of *L*. *casei* were subjected to in vitro simulated physicochemical conditions of the stomach and small intestine; the composition of the digestive juices was set as follows:

In the stomach stage (100 mL): PBS (80 mL), mucin (4 g/L), pepsin (3 g/L), and cow’s milk (20 mL); the pH was adjusted to 2 by the addition of 5 M HCl; the duration of this stage was 90 min.

In the small intestine stage (100 mL): PBS (100 mL), pancreatin (1 g/L), hepatic bile (3 g/L), and mucin (4 g/L), the pH was adjusted to 6.8 by adding 1.5 N NaOH; the duration of this stage was 150 min. During the kinetics of viability loss, samples of 1 mL were taken every 30 min, either free or immobilized cells, to quantify, through dilutions, the number of viable cells in Petri dishes containing MRS culture media and agar, which were incubated for 48 h at 37 ºC. The gastrointestinal simulation was performed nine times.

### Streptozotocin-Nicotinamide-Induced Type 2 Diabetes Mellitus in Rats

We used adult male Wistar rats of 8 weeks and 250 ± 50 g of weight; rodents were purchased from the FES Iztacala-UNAM. All rats were maintained under standard conditions of temperature (22–23 °C), light-dark cycles of 12 h/12 h, conventional food (LabDiet 500I) (Table 1), and purified water ad libitum. The experiments were done under the Official Mexican Standards (NOM-062-ZOO-1996 [17] and NOM-087-SEMARNAT-SSA1-2002 [18]). The National School of Medicine and Homeopathy Ethics Committee of the National Polytechnic Institute of Mexico verified that the study complied with international regulations and standards (approval number: CBE/024/2019).

**Table 1:** Composition of Lab Diet 5001 Rodent Diet

Protein (g/kg)	241
Fat (ether extract) (g/kg)	50
Carbohydrate (nitrogen-free extract) (g/kg)	487
Fiber (crude) (g/kg)	52
Minerals^a^ (g/kg)	69
Vitamins^b^	-
Gross energy (KJ/g)	17.11

^a^Minerals: calcium, 9.5 g/kg; phosphorus, 6.8 g/kg; phosphorus (non-phytate), 42 g/kg; potassium, 12.1 g/kg; magnesium, 2.1 g/kg; sulfur, 3.3 g/kg; sodium, 3.9 g/kg; chloride, 6.4 g/kg; fluoride, 15 ppm; iron, 240 ppm; zinc, 76 ppm; manganese, 70 ppm; copper, 13 ppm; cobalt, 0.91 ppm; iodine, 0.99 ppm; chromium (added), 0.01 ppm; selenium, 0.41 ppm

^b^Vitamins: carotene, 2.3 ppm; vitamin K, 1.3 ppm; thiamin, 16 ppm; riboflavin, 4.7 ppm; niacin, 130 ppm; pantothenic acid, 24 ppm; choline chloride, 2250 ppm; folic acid, 7.1 ppm; pyridoxine, 6.1 ppm; biotin, 0.3 ppm; vitamin B_12_, 51 μg/kg; vitamin A, 18 IU/g; vitamin D_3_ (added), 4.6 IU/g; vitamin E, 42 IU/kg; ascorbic acid, 0.0 g/kg

To induce type 2 diabetes, the rats were deprived of food and water for 12 h. An intraperitoneal injection of nicotinamide (150 mg/kg) (Sigma-Aldrich) was administered to the rats 15 min before inducing type 2 diabetes. After this period, a second intraperitoneal injection of streptozotocin (50 mg/kg) (Sigma-Aldrich) was given [19]. After 72 h, the blood glucose concentration was measured with a glucometer (Optium Xceed); the rats were considered diabetic when the blood glucose concentration was higher than 125 mg/dL. Then, the rats were randomly divided into seven groups to evaluate the antihyperglycemic effect, each of 10 animals. The groups were as follows: group healthy control (H); group diabetic control (D); group diabetic treated daily with the first vehicle (300 μL PBS/day) (DPBS); group diabetic treated daily with a second vehicle (12 microparticles of calcium alginate plus 200 μL PBS/day) (DPBSA); group diabetic treated daily with metformin (100 mg/kg) (DMET); group diabetic treated daily with free cells of *L. casei* (10^12^ CFU in 300 μL PBS/day) using a gastric tube (DFLC); finally, group diabetic treated daily with immobilized cells of *L. casei* (12 calcium alginate microparticles containing a total of 10^12^ CFU in 200 μL PBS/day) using a gastric tube (DILC). After 45 days of treatment and food deprivation for 12 h, the rats were euthanized by CO_2_-induced asphyxia; then, blood, liver, kidney, large intestine, and pancreas samples were collected from the animals.

### Biochemical Analyses

A blood sample was taken through a cardiac puncture with a syringe; the blood was centrifuged at 570 × *g* for 10 min at 4 °C and processed in the San Rafael Laboratory (Mexico City) in Autokem 11-KONTROLab equipment. The glucose concentration was determined. Also, a hepatic profile was performed. The direct, indirect, and total bilirubin were evaluated. The aspartate aminotransferase (AST), the alanine aminotransferase (ALT), and the alkaline phosphatase (ALP) were determined. On the other hand, lipidic profiles (cholesterol (Cl), triglycerides (Tg), high-density lipoprotein (HDL), low-density lipoprotein (LDL), and very low lipoprotein (VLDL)) and renal profiles (urea (Ur) and creatinine (Cr)) were determined as well. Each parameter was evaluated in triplicate.

### Histopathological Analysis

After dissecting the animals, small sections of the different organs, such as the liver, kidney, large intestine, and pancreas, were placed in 10% formaldehyde in PBS solution. Subsequently, samples of 4–6 µm were prepared, stained with hematoxylin-eosin (H&E), and examined by a microscope (×400). The histological samples obtained from all the experimental groups of rats were observed by the microscope set in 5 different sections in triplicate.

### Statistical Analysis

Data statistical analysis was performed in GraphPad PRISM v8.0a software. A *t*-student analysis was carried out for the kinetics of the viability loss of *L. casei*. A one-way analysis of variance (ANOVA) was performed, and a post hoc Bonferroni test (*p* ≤ 0.05) was accomplished when there were significant statistical differences from the evaluation of the antihyperglycemic effect.

## Results and Discussion

### Kinetics of Viability Loss of Free and Immobilized *Lactobacillus casei* Under In Vitro Simulated Physicochemical Conditions of the Stomach and Small Intestine

**Fig. 1:**
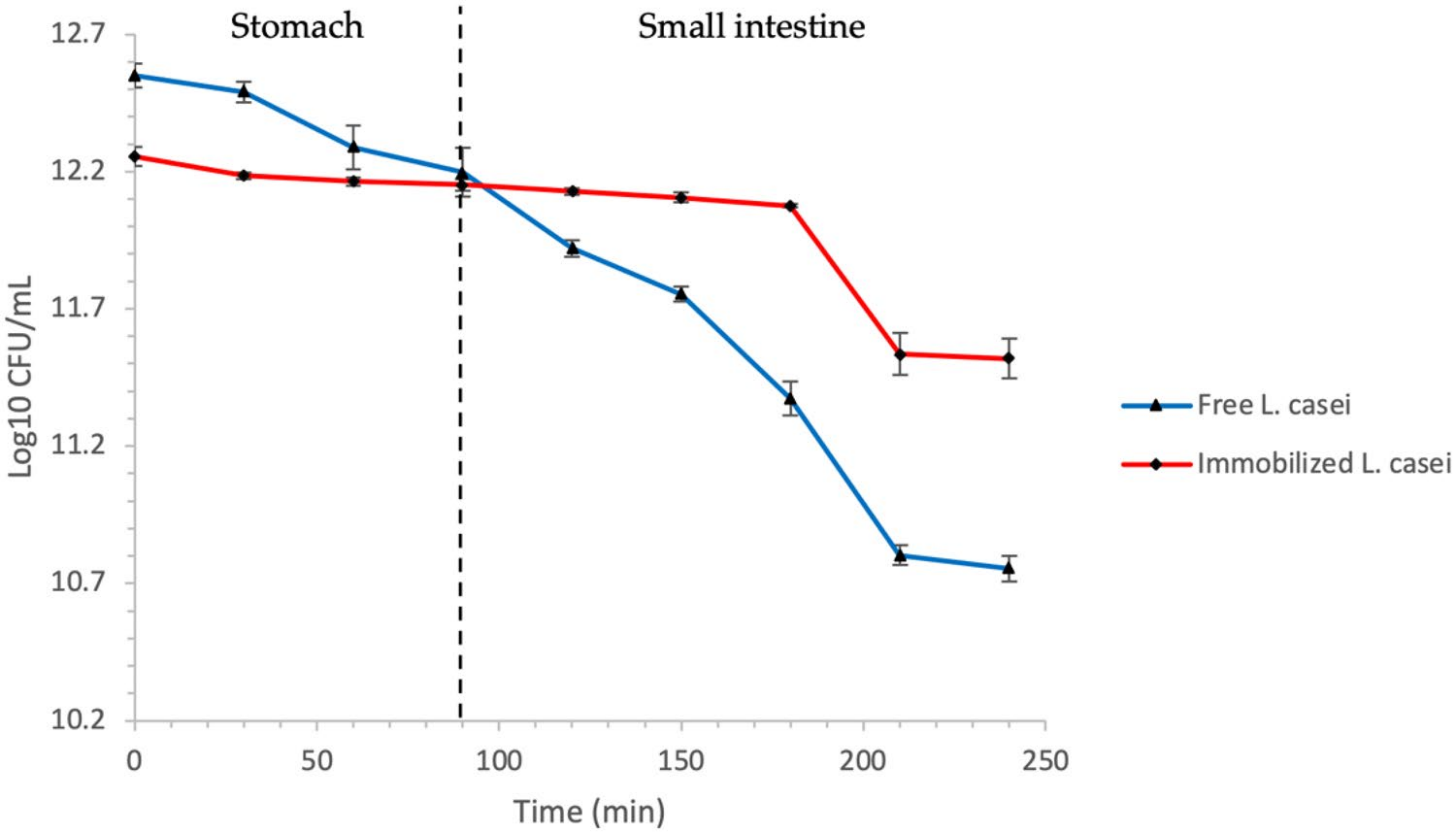
Viability of free and immobilized *L. casei* subjected to in vitro simulated physicochemical conditions of the stomach and small intestine. The results presented are the average of nine repetitions ± the mean standard deviation (*n* = 9)

Free and immobilized cells of *L*. *casei* were subjected for 90 min to in vitro simulated stomach conditions and then subjected to 150 min to in vitro simulated physicochemical small bowel conditions. The results obtained are shown in Fig. 1.

At the beginning of the kinetics, the free and immobilized* L*. *casei* concentration was 12.55 ± 0.04 log^10^ CFU/mL and 12.25 ± 0.03 log^10^ CFU/mL of alginate gel, respectively, which represented 100% of viability. At the end of the stomach stage, it was observed that the viability percentage decreased in both cases. There were 22.57% (11.90 ± 0.11 log^10^ CFU/mL) of free viable cells and 80.83% (12.16 ± 0.01 log^10^ CFU/mL of alginate gel) of immobilized viable cells. At the end of the small intestine stage, only 2.1% of free cells (10.87 ± 0.01 log^10^ CFU/mL) and 51.9% of immobilized cells (11.97 ± 0.002 log^10^ CFU/mL alginate gel) remained viable. While at the end of the viability kinetics, the concentration of *L. casei* viable cells presented statistically significant differences between both treatments.

### Evaluation of the Antihyperglycemic Effect of Free and Immobilized *Lactobacillus casei* in Diabetic Rats

Table 2 shows that after 45 days of treatment, the serum glucose concentration in the DILC group was similar to that of the H and DMET groups. Conversely, the serum glucose concentration was much higher in the D, DPBS, DPBSA, and DFLC groups with significant statistical differences. It is important to note that the group treated with immobilized *L. casei* (DILC group) showed a 67.8% reduction in serum glucose concentration compared to the group treated with free *L. casei *(DFLC group).

**Table 2:** The serum glucose concentration of different rat groups

**Group**	**Glucose (mg/dL)**
Healthy (H)	116 ± 24
Diabetic (D)	461 ± 23^a,b^
Diabetic + PBS (DPBS)	450 ± 9^a,b^
Diabetic + PBS + sodium alginate (DPBSA)	458 ± 14^a,b^
Diabetic + metformin (DMET)	155 ± 22
Diabetic + free *L. casei* (DFLC)	426 ± 73^a,b,c^
Diabetic + immobilized *L. casei* (DILC)	137 ± 36

The results are the average treatments performed in triplicate ± the mean standard deviation, *n* = 3. A one-way ANOVA and Bonferroni’s post hoc analysis *p* ≤ 0.0001 were performed

*H* healthy, *D* diabetic, *DPBS* diabetic + PBS, *DPBSA* diabetic + calcium alginate, *DMET* diabetic + metformin, *DFLC* diabetic + free *L. casei*, *DILC* diabetic + immobilized *L. casei*

^a^Statistically significant differences vs. healthy

^b^Statistically significant differences vs. metformin

^c^Statistically significant differences vs. treated groups

### Hepatic, Lipidic, and Renal Profiles

#### Hepatic Profile

Table 3 shows the data obtained from the hepatic profile. Direct bilirubin presented normal values in groups H and DILC; on the contrary, it increased in groups D, DPBS, DPBSA, DMET, and DFLC. Regarding indirect bilirubin, the H group presented normal values; in contrast, it increased in the groups D, DPBS, DPBSA, DMET, DFLC, and DILC. It is important to note that the increase in indirect bilirubin was less steep in the DILC group. As for total bilirubin, it was found to be normal in the H and DILC groups; in contrast, it increased in the D, DPBS, DPBSA, DMET, and DFLC groups. On the other hand, AST enzyme activity was found to be normal in groups H, DPBS, DMET, and DILC. Particularly, there was an increase in groups D, DPBSA, and DFLC, showing significant statistical differences. ALT enzyme activity was slightly increased in groups H and DILC, while in groups D, DPBS, DPBSA, DMET, and DFLC, enzyme activity was higher, showing significant statistical differences. Finally, the enzymatic activity of ALP showed normal values in groups H and DILC; on the contrary, it increased in groups D, DPBS, DPBSA, and DFLC, showing significant statistical differences.

**Table 3:** Hepatic profile analysis of different rat groups

**Parameter/group**	**Reference values**	**H**	**D**	**DPBS**	**DPBSA**	**DMET**	**DFLC**	**DILC**
Direct bilirubin (mg/dL)	0.03–0.06	0.07 ± 0.02	0.43 ± 0.01^a,b*^	0.26 ± 0.1^a***^	0.14 ± 0.04	0.14 ± 0	0.13 ± 0.04	0.04 ± 0.01
Indirect bilirubin (mg/dL)	0–0.1	0.15 ± 0.05^b*^	1.1 ± 0.01^a,b*^	0.34 ± 0.13^b****^	0.35 ± 0.07^b*^	0.64 ± 0.1^a*^	0.28 ± 0.08^b*^	0.25 ± 0.02^b*^
Total bilirubin (mg/dL)	0.04–0.2	0.22 ± 0.01^b*^	1.6 ± 0^a*^	0.5 ± 0.1^b*^	0.49 ± 0.03^b*^	0.98 ± 0.3^a*^	0.47 ± 0.01^b****^	0.21 ± 0.06^b*^
AST (U/L)	63–175	126 ± 45	326 ± 36^a,b*^	140 ± 19	265 ± 29^a,b***^	160 ± 23	233 ± 9^a,c****^	128 ± 22
ALT (U/L)	17–50	66 ± 27	282 ± 28^a,b*^	126 ± 11	167 ± 74^a,b**^	67 ± 10	259 ± 56^a,b,c***^	54 ± 4
ALP (U/L)	39–216	192 ± 8^b*^	672 ± 225^a*^	797 ± 87^a*^	244 ± 99^b^	754 ± 211^a****^	651 ± 62^a,c*^	168 ± 6^b^

The results are the average treatments performed in triplicate ± the mean standard deviation, *n* = 3. A one-way ANOVA and Bonferroni’s post hoc analysis *p* ≤ 0.0001, *p ≤ 0.0001, **p ≤ 0.12, ***p ≤ 0.0003, and ****p ≤ 0.01 were performed

The reference values were obtained from the Canadian Council on Animal Care Conseil Canadien de Protection des Animaux (CCACCCPA) [20] and Research in Surgery, Supplement [21]. Reference data is shown for evidence of normal values in a healthy individual

*H* healthy, *D* diabetic, *DPBS* diabetic + BPS, *DPBSA* diabetic + calcium alginate, *DMET* diabetic + metformin, *DFLC* diabetic + free *L. casei*, *DILC* diabetic + immobilized *L. casei*

^a^Statistically significant differences vs. healthy

^b^Statistically significant differences vs. metformin

^c^Statistically significant differences vs. treated groups with *L. casei*

#### Lipidic Profile

Table 4 shows the data obtained from the lipid profile analysis. Total cholesterol concentration was found to be normal in groups H, DPBS, DPBSA, and DMET, while there was a slight increase in groups D, DFLC, and DILC. In particular, HDL showed similar concentrations in groups H and D; however, in groups DPBS, DPBSA, DMET, DFLC, and DILC, there was a slight decrease. Regarding LDL, the H, D, DPBS, and DPBSA groups showed very similar concentrations; on the contrary, they increased in the DMET, DFLC, and DILC groups. VLDL showed similar values in the H, DPBS, DMET, and DILC groups. On the other hand, the concentration of VLDL increased in the D, DPBSA, and DFLC groups, with statistically significant differences observed in the DFLC group only. Finally, Tg concentrations were found in similar and normal values in the H, DMET, and DILC groups; they increased in the D, DPBS, DPBSA, and DFLC groups, presenting significant statistical differences.

**Table 4:** Lipidic profile analysis of different rat groups

**Parameter/group**	**Reference values**	**H**	**D**	**DPBS**	**DPBSA**	**DMET**	**DFLC**	**DILC**
**Total cholesterol (mg/dL)**	33–50	40 ± 9	57 ± 4	45 ± 4	37 ± 8	51 ± 7	53 ± 2	57 ± 2
**HDL (mg/dL)**	NR	17 ± 7	16 ± 2	12 ± 2	11 ± 4	11 ± 4	10 ± 2	14 ± 6
**LDL (mg/dL)**	NR	15 ± 3	13 ± 3	16 ± 2	17 ± 1	24 ± 5	23 ± 2	28 ± 7
**VLDL (mg/dL)**	NR	11 ± 3	18 ± 3	15 ± 2	17 ± 4	13 ± 1	21 ± 2^a,b,c*^	12 ± 1
**Triglycerides (mg/dL)**	55–115	58 ± 16	121 ± 39^a,b*^	102 ± 28^a,b**^	139 ± 5^a,b*^	60 ± 7	170 ± 22^a,b,c***^	78 ± 14

The results are the average treatments performed in triplicate ± the mean standard deviation, *n* = 3. A one-way ANOVA and Bonferroni’s post hoc analysis *p* ≤ 0.0001, *p ≤ 0.01, **p ≤ 0.06, and ***p ≤ 0.0004 were performed

The reference values were obtained from the Canadian Council on Animal Care Conseil Canadien de Protection des Animaux (CCACCCPA) [20] and Research in Surgery, Supplement [21]. Reference data is shown for evidence of normal values in a healthy individual

*H* healthy, *D* diabetic, *DPBS* diabetic + BPS, *DPBSA* diabetic + calcium alginate, *DMET* diabetic + metformin, *DFLC* diabetic + free *L. casei*, *DILC* diabetic + immobilized *L. casei*, *NR* not reported

^a^Statistically significant differences vs. healthy

^b^statistically significant differences vs. metformin

^c^statistically significant differences vs. treated groups with *L. casei*

#### Renal Profile

Table 5 reports the data obtained from the renal profile analysis. Urea concentration showed normal values in the H, D, DMET, DFLC, and DILC groups; however, in the DPBS and DPBSA groups, it increased, presenting significant statistical differences. All groups showed normal values regarding creatinine; however, only in the DPBS group was there an increase, showing statistically significant differences.

**Table 5:** Renal profile analysis of different rat groups

**Parameter/group**	**Reference values**	**H**	**D**	**DPBS**	**DPBSA**	**DMET**	**DFLC**	**DILC**
Urea (mg/dL)	68.5–115.8	57 ± 17	95 ± 24^b*^	121 ± 8^a,b**^	106 ± 29^b***^	45 ± 13	69 ± 14	42 ± 15
Creatinine (mg/dL)	0.71–2.29	1.2 ± 0.2	1.6 ± 0.3	1.7 ± 0.1^b^	1.5 ± 0.6	1.1 ± 0.4	0.9 ± 0	0.9 ± 0.2

The results are the average treatments performed in triplicate ± the mean standard deviation, *n* = 3. A one-way ANOVA and Bonferroni’s post hoc analysis *p* ≤ 0.0001, ^*^p ≤ 0.01, ^**^p ≤ 0.1, and ^***^p ≤ 0.02 were performed

The reference values were obtained from the Canadian Council on Animal Care Conseil Canadien de Protection des Animaux (CCACCCPA) [20] and Research in Surgery, Supplement [21]. Reference data is shown for evidence of normal values in a healthy individual

*H*, healthy; *D*, diabetic; *DPBS*, diabetic + BPS; *DPBSA*, diabetic + calcium alginate; *DMET*, diabetic + metformin; *DFLC*, diabetic + free *L. casei*; *DILC*, diabetic + immobilized *L. casei*

^a^Statistically significant differences vs. healthy

^b^Statistically significant differences vs. metformin

### Histological Analysis of Liver, Pancreas, Kidney, and Large Intestine

**Fig. 2:**
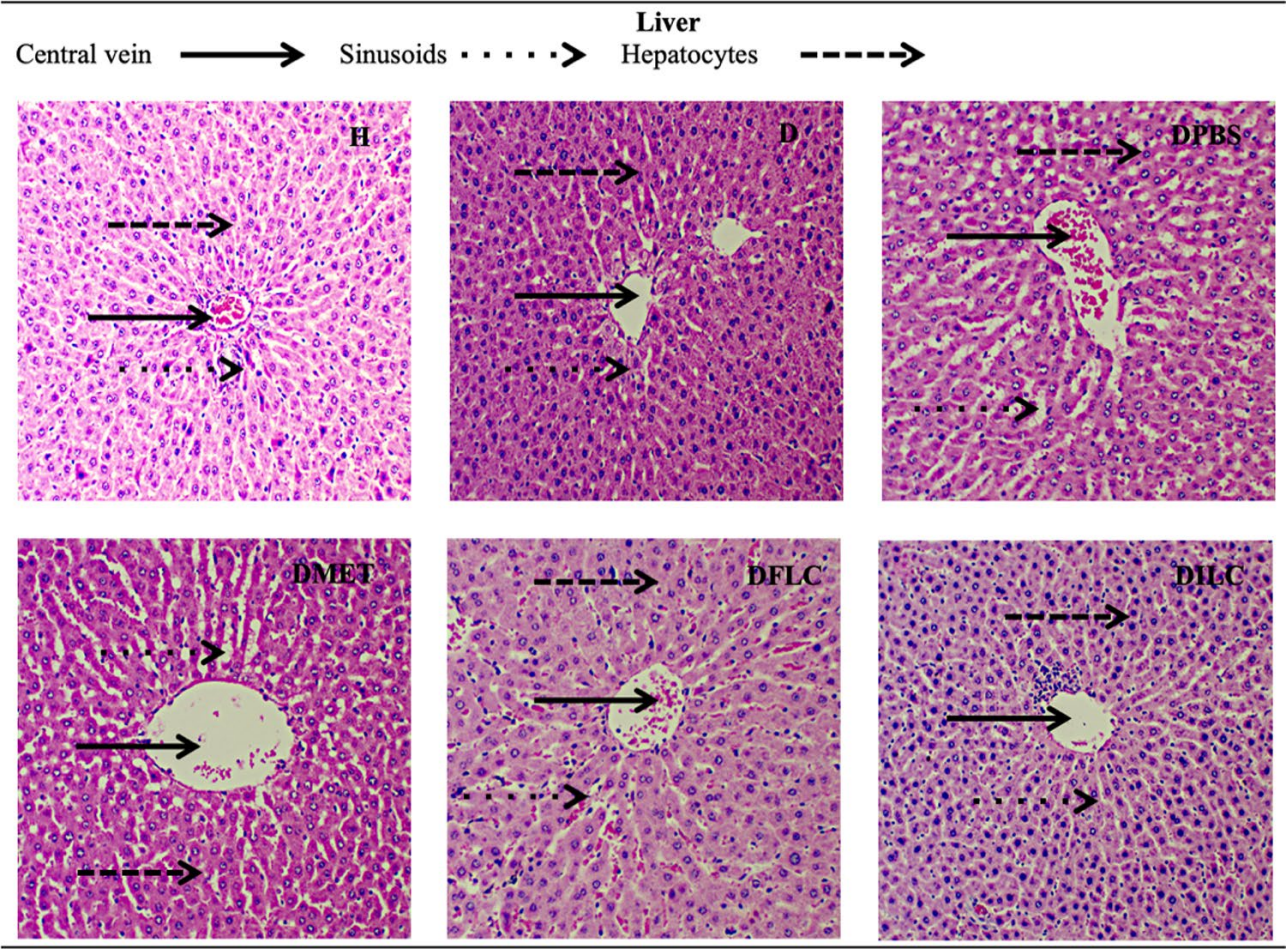
Hematoxylin-eosin staining micrographs of histological liver sections (×400) of different rat groups. H, healthy; D, diabetic; DPBS, diabetic + PBS; DPBSA, diabetic + calcium alginate; DMET, diabetic + metformin; DFLC, diabetic + free *L. casei*; DILC, diabetic + immobilized *L. casei*

A histopathological examination was performed to demonstrate the effect of the consumption of free and immobilized *L. casei* in reducing the organ damage caused by diabetes in rats. Micrographs of the liver, pancreas, kidney, and large intestine were analyzed for the different experimental groups. For instance, Fig. 2 shows micrographs of liver histological sections, showing the central vein, sinusoid, and hepatocytes. In group H, the typical morphology of the liver was observed, while in groups D, DPBS, DPBSA, DMET, and DFLC, there was an increase in the size of the sinusoids; on the contrary, in group DILC, the tissue morphology was very similar to that observed in group H.

**Fig. 3:**
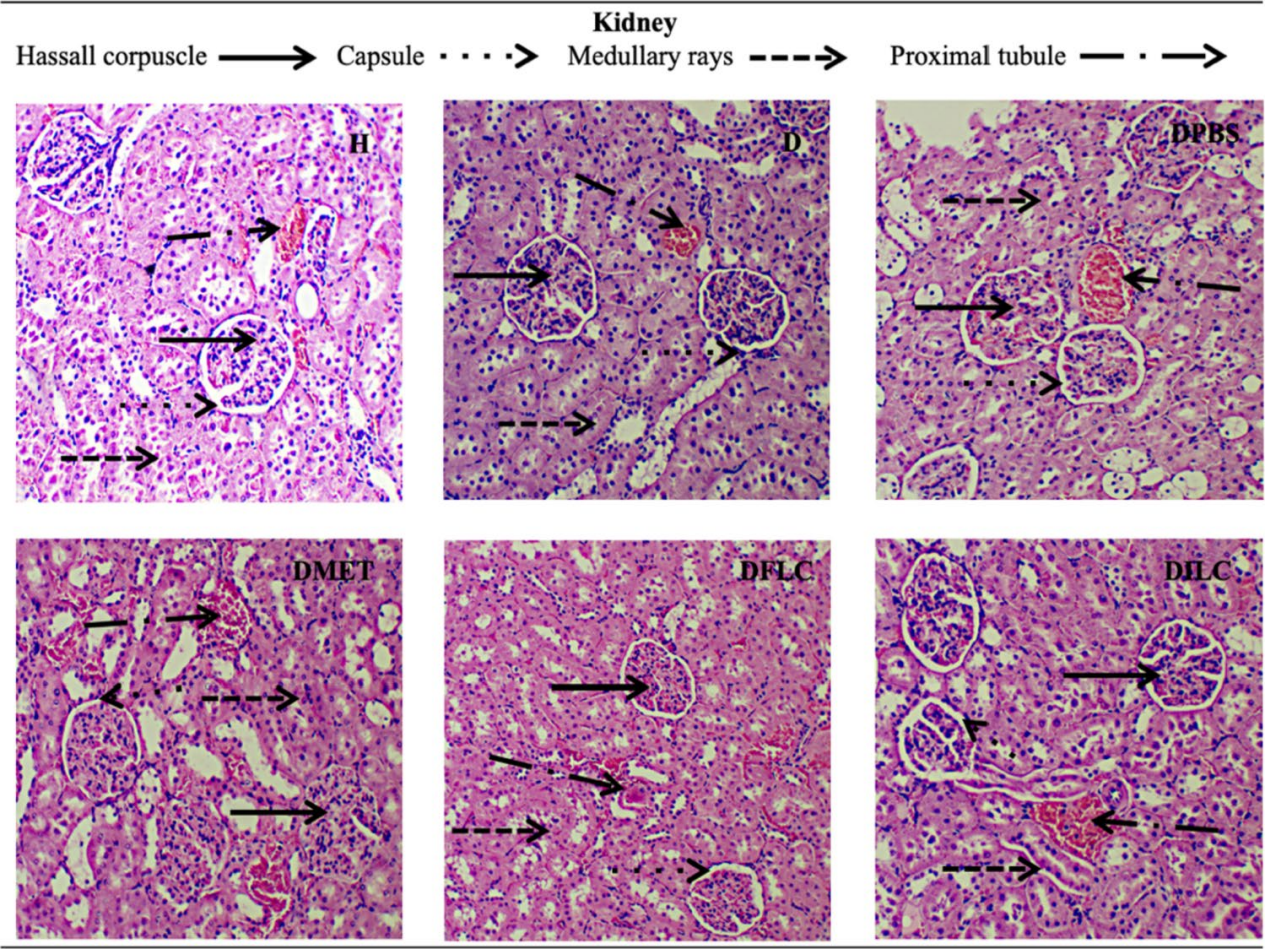
Hematoxylin-eosin staining micrographs of histological kidney sections (×400) of different rat groups. H, healthy; D, diabetic; DPBS, diabetic + PBS; DPBSA, diabetic + calcium alginate; DMET, diabetic + metformin; DFLC, diabetic + free *L. casei*; DILC, diabetic + immobilized *L. casei*

Figure 3 provides micrographs of kidney histological sections where the renal corpuscle, the medullary ray, the proximal convoluted tubule, and the capsule can be observed. In group H, the typical morphology of the kidney was observed. On the other hand, in groups D, DPBS, DPBSA, DMET, and DFLC, deformation of the corpuscles and an increase in the size of the capsule can be seen; on the contrary, in group DILC, the tissue morphology was very similar to that observed in group H.

**Fig. 4:**
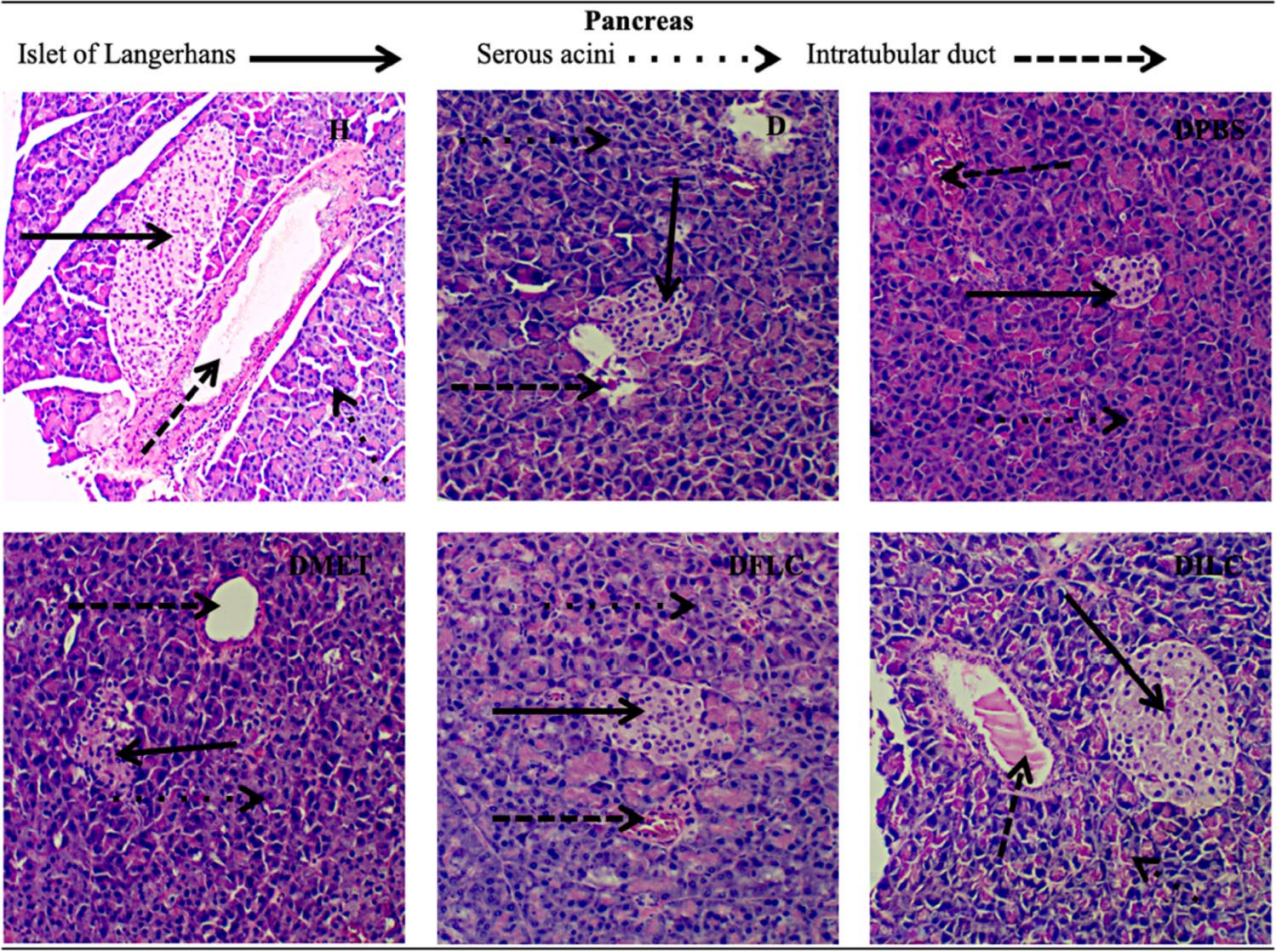
Hematoxylin-eosin staining micrographs of different rat groups’ histological sections of the pancreas (×400). H, healthy; D, diabetic; DPBS, diabetic + PBS; DPBSA, diabetic + calcium alginate; DMET, diabetic + metformin; DFLC, diabetic + free *L. casei*; DILC: diabetic + immobilized *L. casei*

Figure 4 represents micrographs of pancreas histological sections showing the islet of Langerhans, the serous acini, and the intralobular duct. Group H presented a typical pancreas morphology, while groups D, DPBS, DPBSA, and DMET showed small islets and were even broken, so it was difficult to observe them. The islets in the DFLC and DILC groups were similar to those observed in group H.

**Fig. 5:**
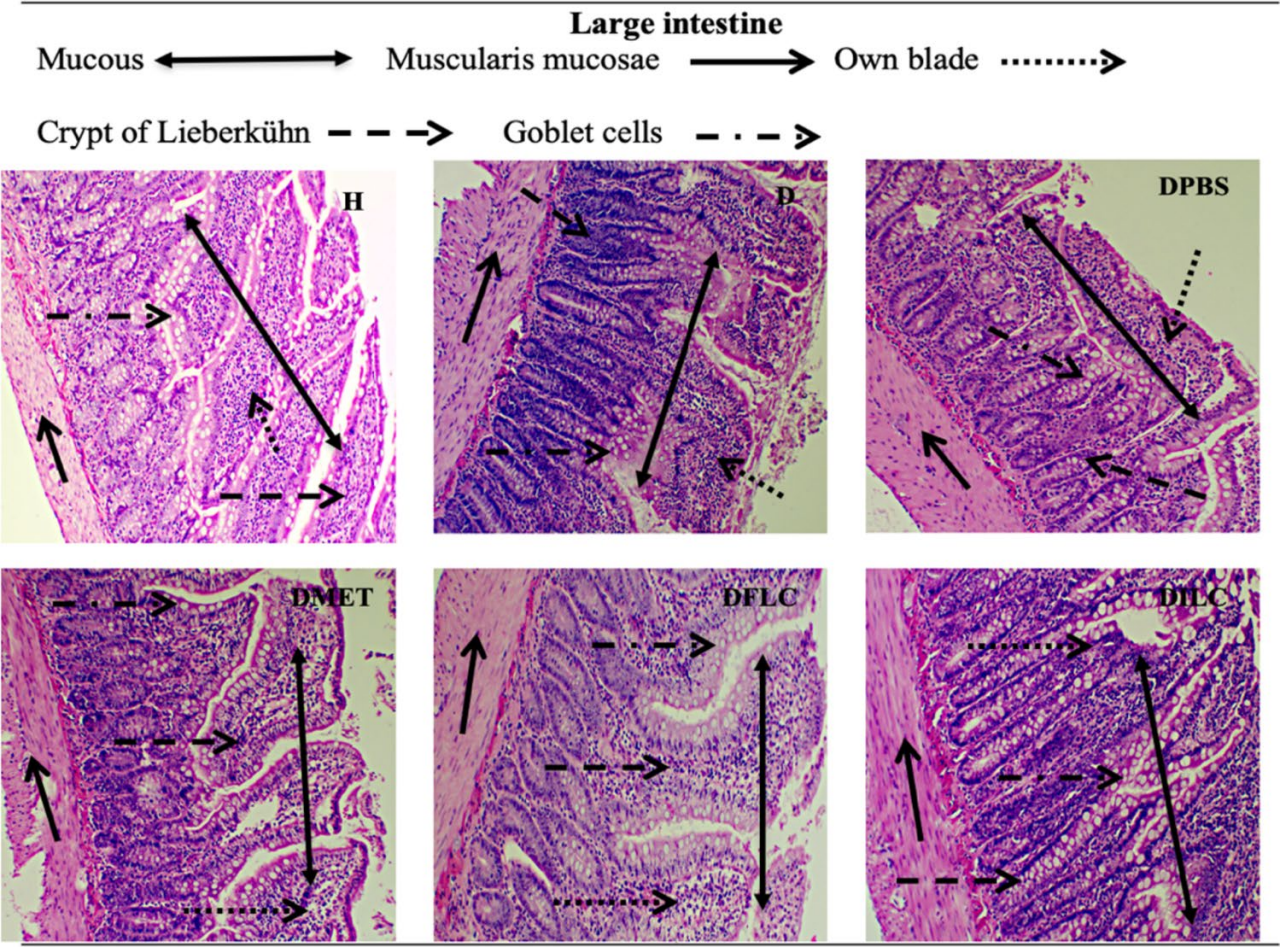
Hematoxylin-eosin staining micrographs of different rat groups’ histological sections of the large intestine (×100). H, healthy; D, diabetic; DPBS, diabetic + PBS; DPBSA, diabetic + calcium alginate; DMET, diabetic + metformin; DFLC, diabetic + free *L. casei*; DILC, diabetic + immobilized *L. casei*

Finally, Fig. 5 shows micrographs of large intestine histological sections. The mucosa, which comprises the lamina propria, the crypt of Lieberkühn, the goblet cells, and the muscularis mucosae, can be observed. In group H, the typical morphology of the large intestine was observed, while in groups D, DPBS, DPBSA, and DMET, there was deformation in the upper part of the crypts and some disintegration. On the other hand, the DFLC and DILC groups presented tissue morphology similar to that observed in group H.

## Discussion

A crucial fact for probiotic bacteria to provide health benefits to the host is their viability at the end of their passage through the digestive system [15, 22, 23]. The cell immobilization approach represents one way to mitigate the damage caused by gastrointestinal physicochemical conditions while preventing the losses of cell viability [13, 14, 22–25]. According to the results, after the in vitro simulation of the gastrointestinal conditions (CGI), the immobilized *L. casei* viable bacteria count was higher than the free form. The recommended daily intake of probiotics is at least 10 log^10^ CFU/mL to 12 log^10^ CFU/mL [26]; thus, cell immobilization meets the recommended probiotic dose. Similar results have been reported using free and immobilized *L. delbrueckii*, *L. acidophilus*, *L. johnsonii*, *L. casei* Shirota, and *L. rhamnosus* [23, 27, 28].

As for dysbiosis, it is defined as an imbalance of the intestinal microbiota, i.e., an increased number of pathogenic microorganisms in the intestines triggers biological processes, which potentiate the development and severity of diabetes [5, 7–9]. In this study, the rat’s intestinal microbiota was selectively modified by consuming free and immobilized *L. casei*. The results demonstrate that the treatments with *L. casei* reduce serum glucose concentration in diabetic rats. However, the decrease in serum glucose levels in diabetic rats treated with free *L. casei* (DFLC group) was only 7.5% compared to the diabetic group (D group), which was not significant; in similar studies carried out with various probiotic strains in free form, the reduction in serum glucose was found to be between 14.8 and 45.4% [29–39]. The serum glucose reduction in the group treated with immobilized *L. casei* (DILC group) was 70.3% compared to the diabetic group (D group). This reduction was significantly higher compared to the treatment with free *L. casei* (DFLC group) and exceeded the outcomes reported in previous studies involving free-form probiotics [3, 30–39]. *L. casei* immobilized showed similar serum glucose concentrations to the healthy group; we associate this behavior to cell immobilization with sodium alginate, as it provides physical protection to the probiotics [23, 24, 40] so that *L. casei* supported the CGI of the rats’ organism. A greater number of viable bacteria effectively carried out their beneficial actions, contributing to the mitigation of hyperglycemia.

To the best of our knowledge, no previous studies have explored the utilization of immobilized probiotics as a supplement for treating diabetes. Therefore, this research represents, for the first time, a pioneering effort to investigate and address this approach. The findings establish that cell immobilization serves as an important vector to protect probiotics from gastrointestinal challenges, enabling them to deliver their therapeutic benefits effectively.

To some extent, the right mechanism declaring probiotic effects in preventing and treating diabetes is unknown so far [41, 42]. However, it has been speculated that probiotic bacteria avoid synthesizing nitric oxide, which causes the formation of free radicals that lead to the deterioration of pancreatic β-cells [41, 43, 44]. On the other hand, Gram-negative bacteria membrane contains lipopolysaccharides (LPS) related to the production of endotoxins capable of crossing the intestinal barrier, causing inflammation and deterioration of the pancreas, liver, and kidney [45]. The consumption of probiotics balances the intestinal microbiota, inhibiting the proliferation of Gram-negative bacteria and reducing the permeability of the intestine [6, 45–50]. Probiotics also produce short-chain fatty acids (SCFA), such as propionate, acetate, and butyrate [3, 25, 30, 44, 45, 51–53], in which the latter compound interacts with intestinal L cells, leading to the overexpression of glucagon-like peptide (GLP-1), which is responsible for stimulating insulin secretion in the pancreas [6, 43, 45, 47, 51, 54–57]. Knowing that there is still some insulin production in DM2, it is likely that this mechanism occurred in our study.

When evaluating the liver profile, bilirubin concentration along with AST, ALT, and ALP enzyme activities, as reported in Table 2, the group treated with free *L. casei* (DFLC group) showed higher values than the group treated with immobilized *L. casei* (DILC group); other authors reported similar results when using probiotics in the free form [31, 33, 39]. This behavior is expected since diabetes is related to liver disease [58, 59]. Moreover, it can be affirmed that liver damage is indirectly reduced by enriching the diet with immobilized *L. casei*.

Concerning the lipid profile, the groups of rats treated with free and immobilized *L. casei* showed changes in lipid metabolism; this behavior has been already observed by several authors [3, 30–32, 37, 39, 43, 48, 60–62]. For instance, these results could be attributed to a metabolic disorder characteristic of diabetes known as dyslipidemia, caused by increased free fatty acids in the body, insulin resistance, and an increase in inflammatory adiposity [63].

We believe that the increase in urea concentration in the group treated with free *L. casei* was due to the high serum glucose concentration leading to the onset of renal damage; other authors have described similar behaviors using free probiotics [31, 32].

Although the group of rats treated with free *L. casei* did not show statistically significant differences in certain evaluated parameters, the results differed from the typical values. An opposite fact was observed in the group treated with immobilized *L. casei*, where only cholesterol was slightly increased. Although there are no reports of similar studies using immobilized probiotics, the data show that treatment with immobilized *L. casei* was more efficient than treatment with free *L. casei*.

As observed in the histopathological analysis, the microscopic morphology of the kidney, liver, pancreas, and large intestine of the DILC group showed no visual differences compared to the H group; similar behaviors have been reported in various studies using free probiotics [37]; however, in our work, the treatment with immobilized probiotics significantly reduced organ damage. In the micrographs of the pancreas, no differences were observed between the DFLC and DILC groups. However, according to the CGI simulations, the viability of free *L. casei* was strongly affected; for this reason, although the islet was observed, there was no significant reduction in the serum glucose concentration.

## Conclusions

The results obtained in this work demonstrate that cell immobilization is an important vector to provide physical protection for probiotics, such as *L. casei*, from simulated gastrointestinal conditions in vitro, and quite possibly in vivo. Interestingly, the consumption of immobilized *L. casei* could allow delivering a high quantity of viable probiotics into the gut, reducing serum glucose concentration by up to 70% compared to diabetic rats and reducing organ damage caused by diabetes. The results also suggest that the consumption of immobilized *L. casei* may help control and treat DM2 by reducing glucose concentration, maintaining biochemical parameters at nominal values, and reducing the damage that DM2 generally causes to organs. However, further investigation into the mechanism of action needs to be addressed. To some extent, all these findings provide a basis for future clinical trials.

## Data Availability

All data analyzed in this paper are already included herein. Any extra data of interest are available from the corresponding author on reasonable request.
